# SEOM clinical guidelines for the treatment of non-small cell lung cancer (NSCLC) 2015

**DOI:** 10.1007/s12094-015-1455-z

**Published:** 2015-12-21

**Authors:** R. García-Campelo, R. Bernabé, M. Cobo, J. Corral, J. Coves, M. Dómine, E. Nadal, D. Rodriguez-Abreu, N. Viñolas, B. Massuti

**Affiliations:** Medical Oncology Department, Complejo Hospitalario Universitario A Coruña, Coruña, Spain; Medical Oncology Department, Hospital Universitario Nstra. Sra. de Valme, Seville, Spain; Medical Oncology Department, Hospital Universitario Málaga, Málaga, Spain; Medical Oncology Department, Hospital Universitario Virgen del Rocío, Seville, Spain; Medical Oncology Department, Hospital Son Llatzer, Palma De Mallorca, Spain; Hospital Universitario Fundación Jiménez Díaz. Oncohealth Institute, Madrid, Spain; Medical Oncology Department, Instituto Catalán de Oncología, L’Hospitalet, Barcelona, Spain; Medical Oncology Department, Hospital Universitario Insular de Las Palmas de Gran Canaria, Las Palmas, Spain; Medical Oncology Department, Hospital Clinic I Provincial de Barcelona, Barcelona, Spain; Medical Oncology Department, Hospital General Universitario Alicante, Alicante, Spain

**Keywords:** NSCLC, Radiotherapy, Chemotherapy, Targeted therapies

## Abstract

Lung cancer is the most common cancer worldwide as well as the leading cause of cancer related deaths as reported by Torre et al (CA Cancer J Clin 65:87–108, 2015]. Non-small cell lung cancer (NSCLC) accounts for up to 85 % of all lung cancers. Multiple advances in the staging, diagnostic procedures, therapeutic options, as well as molecular knowledge have been achieved during the past years, although the overall outlook has not greatly changed for the majority of patients with the overall 5-year survival having marginally increased over the last decade from 15.7 to 17.4 % as reported by Howlader et al.  (SEER Cancer Statistics Review 2015).

## Methodology

Relevant studies published in peer review journals were used for the guideline elaboration. The Infectious Diseases Society of America grading system was used to assign levels of evidence and grades of recommendation.

## Diagnosis

Anatomopathological diagnosis of non-small cell lung cancer should be made according to the World Heath Organization (WHO) classification. The International Association for the Study of Lung Cancer (IASLC) provided adenocarcinoma classification as well as key recommendations for the management of small biopsies and cytology [3]. For therapeutic implications, specific subtyping of NSCLC is strongly recommended whenever possible. A limited diagnostic workup is also recommended to preserve as much tissue as possible for further molecular assessments. Evidence-based recommendations for molecular testing in lung cancer have been recently updated by SEOM–SEAP (Spanish Society of Medical Oncology–Spanish Society of Pathology) [4] (Table 3).

## Staging

In NSCLC the following staging work-up is highly recommended:

- Clinical history, including smoking and family history; physical examination, performance status (PS) and weight loss should be assessed.
- Blood test, including hematology, renal and hepatic function.
- Chest and upper abdomen (including liver and adrenal glands) computerized tomography (CT).
- Brain CT or magnetic resonance imaging (MRI) if there are neurological symptoms in the physical examination.
- Bone scan if there is bone pain, high serum calcium or high alkaline phosphatase.

In patients undergoing potentially radical treatment, the following recommendations should be considered:

- Whole-body FDG-positron emission tomography (PET)-CT
- Bronchoscopy
- Pulmonary function tests
- Ergospirometry if the pulmonary function tests are not normal
- Chest MRI in Pancoast tumour
- Invasive mediastinal staging, endobronchial ultrasound-guided fine-needle aspiration (EBUS-FNA), and/or endoscopic ultrasound guided fine-needle aspiration (EUS-FNA), is recommended in patients with PET positive mediastinal or hilar lymph nodes (LNs). For patients with suspect LNs on imaging and negative EBUS/EUS results, an additional mediastinoscopy is recommended. In patients with PET-negative LNs, invasive staging is also recommended in CT enlarged mediastinal LNs (>1.5 cm) and in patients with central tumours.
- Histological and cytological confirmation is strongly recommended in the presence of pleural/pericardial effusion or isolated metastatic site.

### Staging system

NSCLC is staged according to the UICC system (7th edition), grouped into stage categories (Tables 1 and 2) [5]. The 7th edition is recommended until the 8th will be approved in 2016.

**Table 1:** Staging Grouping (Adapted from Goldstraw et al. [5])

Occult carcinoma	TX	N0	M0
Stage 0	Tis	N0	M0
Stage IA	T1a, b	N0	M0
Stage IB	T2a	N0	M0
Stage IIA	T1a,b	N1	M0
	T2a	N1	
	T2b	N0	
Stage IIB	T2b	N1	
	T3	N0	
Stage IIIA	T1,T2	N2	
	T3	N1,N2	
	T4	N0,N1	
Stage IIIb	T4	N2	
	Any T	N3	
Stage IV	Any T	Any N	M1a,b

**Table 2:** TNM classification 7ª edition (Adapted from Goldstraw et al. [5])

TNM classification	
Primary tumor (T)	
TX	Primary tumor cannot be assessed, or tumor proven by the presence of malignant cells in sputum or bronchial washings but not visualized by imaging or bronchoscopy
T0	No evidence of primary tumor (Tis Carcinoma in situ)
T1	Tumor 3 cm or less in greatest dimension, surrounded by lung or visceral pleura, without bronchoscopic evidence of invasion more proximal than the lobar bronchus (for example, not in the main bronchus) [1]
T1a	Tumor 2 cm or less in greatest dimension
T1b	Tumor more than 2 cm but 3 cm or less in greatest dimension
T2	Tumor more than 3 cm but 7 cm or less or tumor with any of the following features (T2 tumors with these features are classified T2a if 5 cm or less): involves main bronchus, 2 cm or more distal to the carina; invades visceral pleura (PL1 or PL2); associated with atelectasis or obstructive pneumonitis that extends to the hilar region but does not involve the entire lung
T2a	Tumor more than 3 cm but 5 cm or less in greatest dimension
T2b	Tumor more than 5 cm but 7 cm or less in greatest dimension
T3	Tumor more than 7 cm or one that directly invades any of the following: parietal pleural (PL3), chest wall (including superior sulcus tumors), diaphragm, phrenic nerve, mediastinal pleura, parietal pericardium; or tumor in the main bronchus less than 2 cm distal to the carina but without involvement of the carina; or associated atelectasis or obstructive pneumonitis of the entire lung or separate tumor nodule(s) in the same lobe
T4	Tumor of any size that invades any of the following: mediastinum, heart, great vessels, trachea, recurrent laryngeal nerve, esophagus, vertebral body, carina, separate tumor nodule(s) in a different ipsilateral lobe
Regional lymph nodes (N)	
NX	Regional lymph nodes cannot be assessed
N0	No regional lymph node metastases
N1	Metastasis in ipsilateral peribronchial and/or ipsilateral hilar lymph nodes and intrapulmonary nodes, including involvement by direct extension
N2	Metastasis in ipsilateral mediastinal and/or subcarinal lymph node(s)
N3	Metastasis in contralateral mediastinal, contralateral hilar, ipsilateral or contralateral scalene, or supraclavicular lymph node(s)
Distant metastasis (M)	
M0	No distant metastasis
M1	Distant metastasis
M1a	Separate tumor nodule(s) in a contralateral lobe, tumor with pleural nodules or malignant pleural (or pericardial) effusion
M1b	Distant metastasis (in extrathoracic organs)

## Treatment

### Stage I–II

Patients with clinically stage I–II NSCLC should be evaluated in a multidisciplinary tumor board and a preoperative pulmonary assessment is recommended to identify patients at increased risk of post-operative complications following lung cancer.

#### Surgery

Surgical resection is the treatment of choice for patients with early-stage NSCLC, and yields the best potential cure rate in patients with stage I–II and no medical contraindications to operative intervention **(IB).**

The surgical resection used will depend on the extent of the disease, the location of the tumor and the cardiopulmonary reserve of the patient:

- In stage I–II NSCLC patients who are medically fit for surgery, a lobectomy or anatomic pulmonary resection is recommended rather than a sublobar resection **(IB)** [6]. Systematic mediastinal lymph node sampling or dissection at the time of anatomic resection is also recommended for accurate staging over selective or no sampling [7] **(IB)**.
- A sublobar resection (segmentectomy or a non-anatomical wedge resection) is recommended for those patients who cannot tolerate a lobectomy due to comorbidities or decreased pulmonary function **(IB)**.
- A sublobar resection with negative margins can be considered for patients with small peripheral nodules (≤1 cm) with a predominantly ground glass opacity **(IB)**.
- In central tumors, a sleeve lobectomy is the preferred type of resection over a pneumonectomy **(IIC)**.
- Re-resection is recommended for patients with positive margins in resected stage I-II NSCLC. If re-resection is not possible, postoperative radiotherapy (PORT) should be considered [8].

#### Adjuvant therapy

The beneficial effect in terms of survival of adjuvant cisplatin based chemotherapy in completely resected fit stage II–III NSCLC patients is now well established [9].

- For patients with completely resected stage II NSCLC, four cycles of postoperative platinum-based chemotherapy are recommended **(IA)**.
- Postoperative chemotherapy is not recommended for patients with completely resected stage IA NSCLC **(IB)** and its use remains controversial in patients with large IB tumors (≥4 cm) **(IC)**.
- In elderly fit patients (≤80 years), postoperative platinum-based chemotherapy should be considered as well.

*PORT* is not recommended for patients with completely resected stage I–II NSCLC (**IA** and **IIA**, respectively) [10].

#### Stereotactic radiotherapy (SBRT)

SBRT is recommended for patients with node negative tumors ≤5 cm who are deemed medically inoperable or who decline surgery **(IIC)**. Several non-randomized studies suggested that this technique might be a suitable option for operable patients older than 75 years **(IIC)**.

*Targeted agents* are not recommended in the postoperative setting. Adjuvant erlotinib did not improve disease-free survival in patients with EGFR-expressing NSCLC or in the *EGFR* mutant subgroup [11]. Several trials are currently testing the use of targeted therapies in patients with resected *EGFR/ALK* positive NSCLC.

### Stage III

**Fig. 1:**
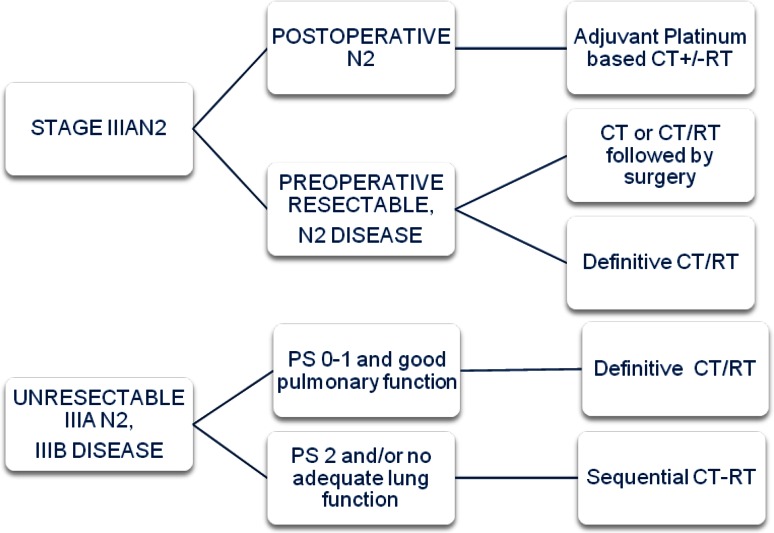
Treatment algorithm for Stage III

Stage III NSCLC represents a heterogeneous group of patients with presentations that range from resectable tumors to unresectable ones. Due to the complexity of most stage III disease presentations, treatment decision must be made within an expert multidisciplinary team management (Fig. 1).

Stage III has been classified into different subgroups:

- In patients with R0 resected NSCLC and an incidental N2 metastases found on final pathology examination of the resection specimen, adjuvant chemotherapy should be given [8] **(IA)**. PORT may be considered (**IVC**) and should be administered after adjuvant chemotherapy. Retrospective analyses from randomized trials suggest a potential benefit of adjuvant radiotherapy in N2 disease. There is an ongoing European trial (LungART) evaluating this strategy.
- In patients with N2 documented intra-operatively, surgery should be followed by adjuvant chemotherapy **(IA)** +/- PORT **(IVC).**
- In potentially resectable IIIA (N2), several randomized clinical trials have compared the outcome of primary surgery versus neoadjuvant therapy followed by surgery with fairly consistent trend to better survival for combined treatment. The Cochrane meta-analysis demonstrated that preoperative therapy is better than surgery alone for patients with stage III [12]. There are also several trials that have evaluated the role of surgery after preoperative therapy compared with a nonsurgical curative-intent strategy obtaining similar results. The North American intergroup 0139 study showed better progression-free survival (PFS) but no survival except in the unplanned subgroup patients who underwent lobectomy [13]. The optimal chemotherapy regimen has not been investigated in randomized studies, but cisplatin-based chemotherapy is recommended. These patients could be treated with induction chemotherapy followed by surgery, induction chemoradiotherapy followed by surgery or concurrent definitive chemoradiotherapy **(IA)**. Trimodality treatment is preferably planned in patients in whom a complete resection by lobectomy is expected.
- In unresectable IIIA (N2) (bulky and multiple mediastinal nodal involvement) and IIIB disease, PS 0-1 and minimal weight loss, concurrent chemoradiotherapy is the treatment of choice **(IA)**. Several phase III trials and a meta-analysis based on individual patient data have showed an overall survival (OS) benefit of 4.5 % at 5 years [14]. For fit patients with inoperable stage III, 2–4 cycles of cisplatin-based chemotherapy is recommended **(IA)**, being etoposide and vinorelbine platinum combinations the most commonly used. There is no evidence for induction or consolidation treatment.
- If concurrent chemoradiotherapy is not possible, induction chemotherapy followed by definitive radiotherapy is an effective alternative [15] **(IA)**.
- Radiotherapy dose of 60–66 Gy in 30–33 daily fractions of 1.8–2 Gy is recommended for concurrent chemoradiotherapy. The RTOG 0617 study has demonstrated that radiation dose of 74 Gy is not superior to the standard dose [16] **(IA)**.

There is no role for prophylactic cranial irradiation in stage III [17] **(IIA)**.

There is currently no role for targeted agents in the treatment of stage III [18] **(IA)**.

### Stage IV (Fig. 2)

#### First line therapy

**Fig. 2:**
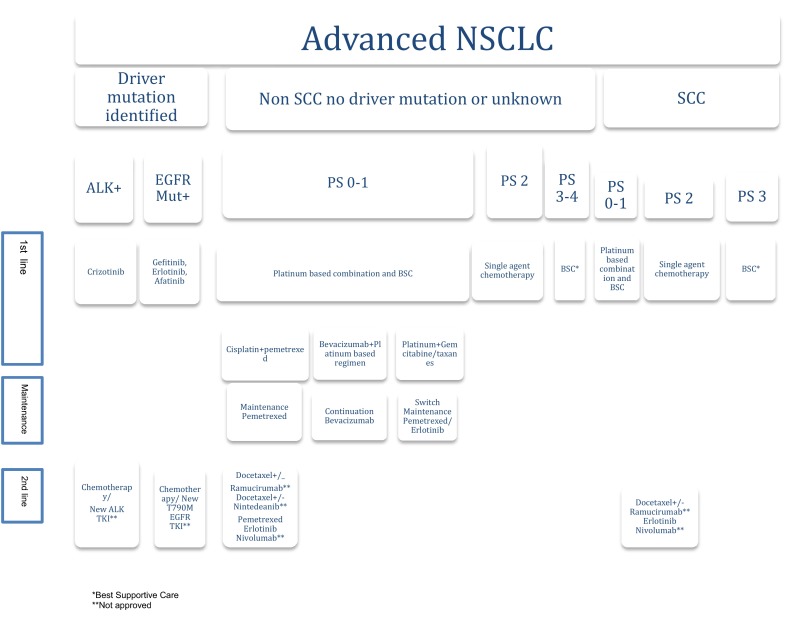
Treatment algorithm for Stage IV

For stage IV PS 0-1 NSCLC patients, without driver mutations, a combination of cytotoxic chemotherapy is recommended, based on tumor histology **(IA).** Early palliative care is strongly recommended (Fig. 2).

##### For squamous cell lung cancer (SCC)

- Two drugs platinum based combination must be offered. Data have shown that platinum combination therapy increases OS and improves QoL compared to supportive care [19] **(IA)**. None of the cisplatin or carboplatin regimens with third generation drugs have shown clear superiority over others in the treatment of SCC. The choice of the combination must take into account the toxicity profile and patient comorbidities **(IA)**.
- Although cisplatin and carboplatin have demonstrated similar activity, a meta-analysis has reported higher response rate (RR) and significantly OS increase in patients treated with cisplatin [20]. Carboplatin can be recommended if any contraindications for cisplatin exist **(IA)**.
- The non-platinum regimens have reported lower activity as compared to platinum regimens [21] **(IB**).
- Four, up to a maximum of six cycles in selected cases, are recommended [22] **(IA)**.

##### For non-squamous cell lung cancer (Non-SCC)

- Platinum-doublet combination is also recommended. Cisplatin/pemetrexed demonstrated more efficacy and less toxicity compared to cisplatin/gemcitabine [23] **(IA)**. If any contraindications for cisplatin exist or in elderly fit patients, the combination pemetrexed/carboplatin could be a valuable treatment option [24] **(IB)**.
- Bevacizumab can be added to the first line treatment in combination with platinum regimens in patients with PS 0-1 and without any specific contraindication for antiangiogenic therapy **(IA)**. Bevacizumab must continue to be administered until disease progression or toxicity [25].
- Non-platinum combinations can be considered in some cases.
- Chemotherapy should be continued for a total of 4–6 cycles in selected cases.
- Maintenance therapy can be considered in those PS 0-1 patients who achieve at least stabilization and have recovered from toxicities from the previous induction therapy **(IA**):
- Pemetrexed and erlotinib can be used as switch maintenance after four cycles of platinum-base induction chemotherapy [26, 27] **(IIB)**.
- Pemetrexed is also indicated in continuation maintenance after four induction cycles of platinum/pemetrexed [28] **(IA)**.
- Maintenance should be administered until unacceptable toxicity or disease progression.

Elderly and PS2:

- Elderly fit patients with PS 0-1 should be treated with platinum combination chemotherapy according to histology [29] **(IA)**.
- Patients with important comorbidities or PS2 are suitable for being treated with monotherapy regimen.
- Unfit PS 3-4 patients, should not receive chemotherapy regardless of age, and supportive care must be recommended. However, patients with *EGFR* mutations or *ALK* rearrangements may also be offered an *EGFR* or *ALK* TKI **(IIA)**.

##### Solitary metastases

Patients with limited disease in chest and unique metastasis site (mainly solitary brain or adrenal metastasis) may benefit from an aggressive local therapy approach (RT, surgery or SBRT) both in the primary and metastatic site [30] **(IIIA)**.

#### Second and third line

- Patients clinically or radiologically progressing after first-line chemotherapy, PS 0-1 should be offered second-line treatment **(IA)**.
- Docetaxel, erlotinib, or pemetrexed (only in non-SCC) have demonstrated improvement in terms of OS and QoL [31–33] **(IA)**.
- Combination regimens have failed to show any survival benefit over single agents with more toxicity [34] **(IA)**.
- Erlotinib may be recommended as third-line therapy for patients with PS of 0-2 who have not received prior EGFR TKIs **(IA)**.
- Recently, novel therapeutic strategies have demonstrated significant benefit in OS in the second line setting, but they have not been approved yet by the Spanish Agency of Drugs and Sanitary Products (AEMPS):
  - The addition of ramucirumab [35] (monoclonal antibody against *VEGFR*-*2*) to docetaxel, demonstrated a significant OS benefit compared to docetaxel alone in previously treated PS 0-1 NSCLC patients.
  - Nintedanib [36] (*VEGFR 1*-*3, FGFR 1*-*3, PDGFR alpha/beta* and *RET* TKI) added to docetaxel has demonstrated a significant OS benefit as compared with docetaxel alone in previously treated stage IV, PS 0-1 adenocarcinoma.
  - Nivolumab, *PD*-*1* monoclonal antibody, improves the RR and the OS as compared with docetaxel alone in previously treated SCC NSCLC independently of the *PD*-*L1* expression [37]. Preliminarily, Nivolumab resulted no survival inferior to docetaxel in the non-SCC population, but improved overall RR and OS in patients with *PD*-*L1* overexpression [38].

#### Targeted therapy for stage IV NSCLC

Significant advances in understanding the molecular biology of NSCLC led to the identification of driver alterations and novel therapeutic targets. The majority of these alterations occur in adenocarcinomas although potential targets in SCC are also emerging. Drugs targeting the *EGFR* and *ALK* genes, respectively, are currently approved. Testing for both alterations is recommended upfront in stage IV non-SCC regardless of clinical characteristics, and in non-smokers irrespective of histology **(IA).**

#### *EGFR* mutation

The frequency of *EGFR* mutations in Spanish population is around 10–16 % of patients. The most commonly found *EGFR* mutations are the exon 19 deletions (Del19) and the exon 21 L858R point mutation (85–90 %).

First-line patients harboring *EGFR* mutations should be treated with an *EGFR* TKI (Gefitinib, Erlotinib, Afatinib) **(IA)**. Consistent evidence of several phase III trials have showed superior PFS, RR, toxicity profiles and QoL for *EGFR* TKIs compared with platinum-based doublets [39–41]. These studies did not show statically significant differences in OS, although a prespecified sub-analysis of LUX-Lung 3 and 6 trials showed a significant improvement in OS favoring afatinib vs chemotherapy in Del19 patients [42]. The results from direct comparative trials among different *EGFR* TKIs are not yet available, although ongoing randomized trials, comparing first generation with second and third generation *EGFR* TKIs are awaited.

For patients with stage IV NSCLC harboring *EGFR* mutations that progressed to first line therapy:

- Rebiopsy is at the time of progression in patients with *EGFR* mutations treated with first- or second-generation *EGFR TKIs* at front line, and benefits/risks should be discussed with the patient **(IIIC)**.
- An *EGFR TKI* should be recommended if not received during the first line setting **(IA)**.
- Platinum-based chemotherapy can be recommended after progression to an *EGFR* TKI **(IIA)**.
- *EGFR* T790M gatekeeper mutation is considered to be the main mechanism of acquired resistance to EGFR TKIs. Third generation *EGFR* TKIs such as AZ9291, Rociletinib, HM61713, EGF 816 or ASP 8273 are selective for T790M resistance mutation and have shown significant activity in several phase I and II trials in patients with acquired resistance to first and second generation *EGFR* TKIs. Results from ongoing clinical trials are awaited to recommend these drugs in the second line setting.
- Continuing *EGFR* TKI in combination with platinum-based chemotherapy beyond progression has failed to demonstrate a significant benefit and should not be recommended [43].

#### ALK gene rearrangement

*ALK* rearrangements, mainly translocations, occur in around 4 % of NSCLC.

- For those *ALK* positive patients, Crizotinib should be recommended in the first line setting **(IA)**. The phase III trial PROFILE 1014, compared crizotinib vs platinum-pemetrexed confirming a significant benefit in terms of PFS, RR and QoL [44].
- If not received during the first line setting, Crizotinib should be recommended as second-line treatment **(IA)**. The recommendation is based on the phase III trial PROFILE 1007, that compared crizotinib vs chemotherapy (either pemetrexed or docetaxel) in patients with locally advanced or metastatic *ALK* positive NSCLC previously treated. Crizotinib achieved significant better outcome in terms of PFS, RR, toxicity profile and QoL [45].
- For those patients progressing on Crizotinib treatment, Ceritinib, a second generation *ALK* TKI has received the approval from the FDA and EMA on the basis of a phase I single-arm trial obtaining a RR of 56 % and mPFS of 6.9 months [46] **(IIB).**
- Chemotherapy may still be appropriate in the absence of phase III data comparing ceritinib with chemotherapy. The chemotherapy regimens are the same as were recommended as first-line using platinum-based combinations **(IA)**.
- Other *ALK* inhibitors under investigation include alectinib, brigatinib and lorlatinib have been reported to have high activity in *ALK* positive patients including patients with brain metastases. Results from ongoing clinical trials evaluating new *ALK* TKIs are awaited.

## Other targetable genetic alterations

The prevalence of other molecular alterations with potentially actionable drugs is low (<2 %). None of these targeted drugs has regulatory approval. Routine testing for these biomarkers is not currently recommended **(IIIC)**.

Early clinical trials have shown the activity of targeting drugs as crizotinib (*ROS1* fusion and *MET* amplification), vemurafenib and dabrafenib or dabrafenib plus trametinib (*BRAF* mutations), anti-HER2 monoclonal antibodies or HER2 TKIs (*HER*2 mutations) and other potential drugs targeting *RET* fusion, *PI3* *K* mutations and others. However, no active agent has been clinically proven yet in *KRAS* mutations. Available evidence for the use of these agents is limited based in early clinical trials (Table 3).

**Table 3:** Summary of recommendations

	Recommendations
**Diagnosis**	
	Pathological diagnosis should be made according to the WHO classification and IASLC classification of adenocarcinoma For therapeutic implications, specific subtyping of NSCLC is strongly recommended whenever possible Limited panel of immunohistochemistry markers is strongly recommended in order to preserve as much tissue as possible for further molecular assessments Testing for EGFR mutations and ALK translocations are recommended in all patients with advanced-stage non-SCC, regardless of clinical characteristics and in never smokers irrespective of histology
**Stage I–II**	
Patients medically fit for surgery	Lobectomy plus systematic lymph node sampling or dissection
Patients medically inoperable, node negative, tumors < 5 cm	SBRT
Post-operative radiotherapy (PORT)	Not indicated in completely resected stage I-II
Adjuvant chemotherapy (four cycles of adjuvant cisplatin-based chemotherapy	Not indicated in stage IA May be considered in selected patients with stage IB Recommended in stage II
Targeted agents	Not recommended
**Stage III**	
Postoperative IIIA (N2)	Adjuvant platinum-based chemotherapy ± PORT
Preoperative resectable IIIA (N2)	Definitive concurrent chemo/radiotherapy Induction chemotherapy or induction chemoradiotherapy followed by surgery evaluation
Unresectable IIIA (N2), IIIB	PS 0-1: definitive concurrent chemoradiotherapy PS 2: sequential chemoradiotherapy
**Stage IV without driver mutations**	
First line setting For PS 0-1, platinum-based doublets are recommended based on tumor histology	
Non-SCC	Platinum-based doublet Cisplatin/pemetrexed doublet has demonstrated more efficacy and less toxicity compared to cisplatin/gemcitabine Bevacizumab added to a platinum doublet if there are no contraindications. Bevacizumab must continue to be administered until disease progression or toxicity
SCC	Platinum-based doublet
Elderly	Elderly fit patients with PS 0-1 should be treated with platinum combination chemotherapy according to histology
PS 0-2	Patients with important comorbidities or PS2 are suitable for being treated with monotherapy regimen
Maintenance	For PS 0-1, non-SCC patients with stable disease or response after four cycles Pemetrexed or erlotinib can be used as switch maintenance Pemetrexed is also indicated in continuation maintenance after four induction cycles of platinum/pemetrexed
Second line setting and beyond	For PS 0-2, docetaxel, erlotinib, or pemetrexed (only in non-SCC) Erlotinib may be recommended as third-line therapy for patients with PS of 0-2 who have not received prior EGFR TKIs
**Stage IV** ***EGFR*** **Mut NSCLC**	
First-line stage IV *EGFR* Mut NSCLC	Gefitinib, erlotinib, afatinib
*EGFR* Mut patients who have not received and EGFR TKI as first line	Gefitinib, erlotinib, afatinib
*EGFR* Mut patients who progressed after first-line treatment with an EGFR TKI	Platinum-based chemotherapy Clinical trials with EGFR T790M TKIs* are ongoing
**Stage IV** ***ALK*** **rearranged NSCLC**	
First-line ALK-rearranged stage IV NSCLC	Crizotinib
Second line *ALK*-rearranged naive patients	Crizotinib
Crizotinib-naive *ALK*-rearranged NSCLC patients who have received one prior platinum-based regimen	Crizotinib
*ALK*-rearranged NSCLC patients who have received previously crizotinib	Chemotherapy Ceritinib*
**Other genetic alterations**	
*Ros1*	Crizotinib* (IIIC)
*Met* amplification	Crizotinib* (IVC)
*BRAF* mut	Vemurafenib*, Dabrafenib* (IVC) Dabrafenib* + Trametinib* (IIIC)
*Her2* mut	Her2 monoclonal antibodies*, Her2 TKIs* (IVC)

* Not approved

## Follow-up

Follow-up frequency in patients with NSCLC is a controversial issue.

After curative-intent

- In patients who have had surgery, follow-up visit including history, physical examination and spiral chest CT is recommended every 6–12 months for the first 2 years and annually thereafter **(IIIB)**.
- For patients who have undergone curative-intent therapy, routine surveillance with blood test, PET imaging or another radiological assessment is not recommended **(IID)**.
- For patients treated with SBRT, CT scans every 6 months for 3 years are recommended if patients are suitable for salvage treatment **(IIIB)**. The use of FDG-PET (and biopsy if positive) is recommended when recurrence after SBRT is suspected based on chest CT **(IIIB)**.

In advanced disease patients:

- Treatment response is recommended to be evaluated 9 or 12 weeks after treatment begins, using the same radiographic method used at baseline. Depending on individual clinical judgement, a repeat scan might be performed after 6 weeks.
- For patients eligible for active cancer therapy in successive lines of treatment, it is advisable to undergo clinical and/or radiological evaluation 6 weeks after finishing treatment and then every 6–12 weeks to enable second-line therapy to commence promptly **(IIIB).**
